# Radiotherapy or chemotherapy: a real-world study of the first-time relapsed and refractory primary central nervous system lymphoma

**DOI:** 10.3389/fonc.2023.1098785

**Published:** 2023-04-27

**Authors:** Yu Yang, Qing Li, Jingjing Ma, Hui Kang, Zhiguang Lin, Yang Wang, Yan Ma, Bobin Chen

**Affiliations:** ^1^Department of Hematology, Huashan Hospital, Shanghai Medical College, Fudan University, Shanghai, China; ^2^Department of Radiology, Huashan Hospital, Shanghai Medical College, Fudan University, Shanghai, China

**Keywords:** relapsed or refractory primary central nervous system lymphoma, radiotherapy, chemotherapy, prognosis, salvage therapy

## Abstract

**Background:**

Primary central nervous system lymphoma (PCNSL) is an uncommon variant of non-Hodgkin lymphoma (NHL) with high aggressiveness and poor prognosis. Although complete remission (CR) could be achieved with therapy, some patients remain refractory or recurrently with a worse response to salvage treatment and poor prognosis. No consensus on rescue therapy has been established currently. This study is aimed to evaluate the efficacy of radiotherapy or chemotherapy in first-time relapsed or refractory progressed PCNSL (R/R PCNSL) and analysis the prognostic factors, to explore differences between relapsed and refractory PCNSL.

**Methods:**

Totally 105 R/R PCNSL patients from Huashan Hospital between 1 January 2016 and 31 December 2020 were enrolled, underwent salvage radiotherapy or chemotherapy and received response assessments after each course. PFS1 was defined as the time from diagnosis to the first time of recurrence or refractory progression. Statistical analysis was performed with SPSS version 26.0.

**Results:**

Response and survival were analyzed over a 17.5months (median) follow-up. Compared to relapsed PCNSL (*n* = 42), refractory PCNSL (*n* = 63) had a shorter median PFS1 related to deep lesions. 82.4% of cases were discovered as the second relapse or progression. ORR and PFS were both higher in relapsed PCNSL than those in refractory PCNSL. ORR of radiotherapy in both relapsed and refractory PCNSL was higher than that of chemotherapy. Elevated CSF protein and ocular involvement were related to PFS and OS after recurrence respectively in relapsed PCNSL. Age ≥ 60y was unfavorable to OS-R (OS after recurrence or progression) in refractory PCNSL.

**Conclusions:**

Our results indicate that relapsed PCNSL responds well to inducing and salvage therapy and has a better prognosis compared to refractory PCNSL. Radiotherapy is effective for PCNSL after the first relapse or progression. Age, CSF protein level, and ocular involvement could be potential factors to predict prognosis.

## Introduction

Primary central nervous system lymphoma (PCNSL) is a rare type of extranodal non-Hodgkin lymphoma (NHL), accounting for less than 3% of NHL and about 2%-4% of primary intracranial tumors (1, 2). PCNSL originates in the central nervous system, with lesions confined to the brain parenchyma, soft meninges, cerebrospinal fluid (CSF), spine, and eyes, and rarely involved other systems.

PCNSL is prevalent in the elderly population over 60 years of age, and a rising incidence has been recognized over the past two decades, reaching 0.5 per 100,000 person-years (2–4). Compared with system lymphomas outside the CNS, the prognosis for PCNSL is usually poor, with a 5-year survival rate of only 30-40%, a median progression-free survival (PFS) of 24 months, and a median overall survival (OS) of 36.9-46 months (5–7). Nearly a century of clinical experience and research have proven that the first-line treatment for newly-diagnosed PCNSL patients is chemotherapy based on high-dose methotrexate (HD-MTX) (>3g/m^2^). However, there are still 10%-35% of refractory PCNSL remain insensitive to HD-MTX, and even among patients who achieve remission with first-line therapy, 35%-60% eventually experience relapse (8). Moreover, the prognosis for PCNSL that has failed first-line therapy remains even worse, although new therapeutic approaches have improved survival (9, 10).

Many studies have been conducted nationally and internationally on salvage therapy for recurrent or refractory PCNSL (R/R PCNSL), but most of them have focused only on either recurrent or refractory PCNSL, or have discussed both groups simultaneously. However, there are significant differences in the overall outcomes of the two groups, and the choice of salvage treatment options is also focused on differently. Therefore, there is still some heterogeneity between the two groups of patients, and the salvage treatment options and the evaluation of their efficacy cannot be generalized.

Thus, the purpose of this study was to compare the efficacy of chemotherapy and radiotherapy for patients with recurrent or refractory PCNSL after the first time of relapse/progression and explore the prognostic factors of R/R PCNSL.

## Methods

### Study design

This retrospective study involved 105 patients with relapsed or refractory PCNSL admitted to Huashan Hospital, Fudan University between 1 January 2016 and 31 December 2020, and was approved by the ethical review boards of Huashan Hospital (KY2017-014). All participants provided informed consent before enrollment.

Relapsed PCNSL was defined as the re-emergence of a new lesion in a patient with PCNSL after achieving CR. Since there was no uniform definition currently, in this study refractory PCNSL was defined as failing to achieve PR after 3 courses or developing PD in 2 courses, referring to clinical experience and diagnostic criteria of other hematologic malignancies (11, 12). The specific evaluation criteria were based on the Lugano criteria for malignant lymphoma (13).

After being evaluated as the first-time relapse or refractory PCNSL, the patient accepted comprehensive evaluation, including but not limited to whole-body positron emission tomography/computed tomography (PET-CT), cranial enhanced magnetic resonance imaging (MRI) with gadolinium enhancer, chest and abdominal CT, ultrasound of superficial lymph nodes, bone marrow examination (smear and biopsy), blood tests, as well as cerebrospinal fluid examination and ophthalmologic examination. Patients with other malignancies, contraindications to radiotherapy or chemotherapy, or active Hepatitis B or C had been excluded.

**Figure 1:**
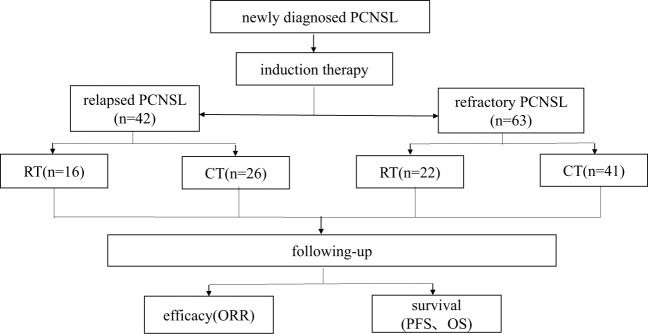
The flowchart of this study.

After being recruited, patients continued to be divided into radiotherapy with or without adjuvant chemotherapy group (hereinafter referred to as radiotherapy group, RT group) and chemotherapy alone group (hereinafter referred to as chemotherapy group, CT group) according to the salvage treatment option chosen after the first relapse/progression. Patients had response assessments after each course. The study process is shown in **Figure 1**. Near-term response to salvage therapy was measured at 1 month after radiotherapy or in 3 courses of chemotherapy. The primary endpoints were overall response rate (ORR) and progression-free survival (PFS). The secondary endpoints were overall survival (OS) and OS after recurrence or progression (OS-R). PFS1 was defined as the time from diagnosis to the first time of recurrence or refractory progression.

### Statistic methods

SPSS 26.0 was used for statistical analysis. The t-test of independent samples and the chi-square test were used to compare differences in measurement data or categorical data between groups, respectively. The Mann-Whitney U test was used to compare nonparametric variables between the 2 groups. Log-rank test and Cox regression model were used to analyze survival data. Variables with p-value < 0.2 were included in multivariate analysis as potential prognostic factors. Data with a p-value < 0.05 are considered statistically significant.

## Results

### Patient characteristics

A total of 105 patients with R/R PCNSL were enrolled and evaluated, with 42 in the relapse group (RL) and 63 in the refractory group (RF). The age at diagnosis was 56 years, 53 years for RL, and 58 years for RF. The study groups were well-balanced in patients and tumor characteristics (**Table 1**). Different induction treatments were performed before this study. The distribution of induction treatment is summarized in **Supplemental Table 1**.

**Table 1:** Clinical and tumor characteristics.

Characteristics	All (*n*)	RL (*n*)	RF (*n*)	*χ^2^*	*p*
Gender					
Male	62	27	35	0.794	0.373
Female	43	15	28		
Median age					
≥60 y	44	14	30	2.113	0.146
<60 y	61	28	33		
Pathology					
DLBCL	93	36	57	3.065	0.216
B-cell lymphoma	10	4	6		
NHL	2	2	0		
KPS					
≥70	58	23	35	0.006	1.000
<70	47	19	28		
Deep lesions					
Present	70	24	46	2.857	0.097
Absent	35	18	17		
Ocular lymphoma^*^					
Present	14	8	6	1.754	0.244
Absent	86	33	53		
Biopsy					
Resection	36	16	20	1.669	0.434
Puncture	67	26	41		
CSF	2	0	2	

*Five patients failed to receive ocular examinations because of poor condition or consciousness disorders.

### Differences of PFS1 in R/R PCNSL

**Figure 2:**
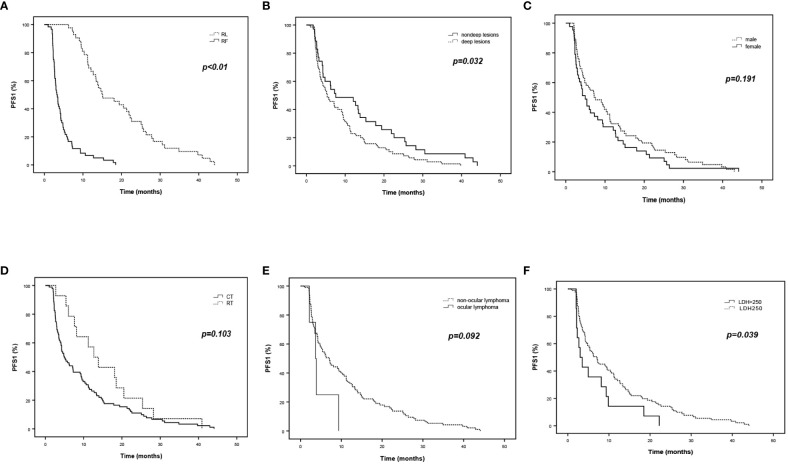
Survival curves of PFS1 in R/R PCNSL. **(A)**. PFS1 (the time from diagnosis to the first time of recurrence or refractory progression) of the RL and RF group. B-F. Prognostic factors for initial recurrence or progression according to PFS1. **(B)** Position of lesions. **(C)** Gender. **(D)** Induction therapy. **(E)** Ocular lymphoma. **(F)** Level of serum LDH.

Since all R/R PCNSL patients had at least one recurrence/progression event, we defined the time from diagnosis to the first time of recurrence or refractory progression as PFS1. The median PFS1 of all recruited patients was 6.2 months (*95% CI*, 4.1 to 8.3), 14.9 months in the RL group, and 3.4 months in the RF group (*95%CI*, 9.0 to 20.8; *95% CI*, 2.8 to 4.0, respectively, *p* < 0.01) (**Figure 2A**). In the RL group, there were 13 patients with PFS1 ≤ 12 months and 29 patients with PFS1 > 12 months. No patients relapsed in 6 months. In the RF group, there were 4 patients with PFS1 > 12 months and 59 patients with PFS1 ≤ 12 months, including 52 patients who had PFS1 ≤ 6 months. The difference proportion of PFS1 ≤ 12 months in the two groups differed significantly (*χ^2 ^= *45.967, *p* < 0.01).

### Prognostic factors of PFS1

**Table 2** and **Figures 2B–F** show multivariate analysis and survival curves of prognostic factors for initial recurrence or progression. Univariate analysis suggested patients with deep lesions are more likely to acquire shorter PFS1 (5.4m vs 7.6m, *p* = 0.032). The HR for PFS1 in all recruited patients with R/R PCNSL was 1.62 for deep lesions compared to non-deep lesions in cox regression analysis (*95%CI*, 1.050 to 2.499, *p* = 0.029). However, no significant independent factors were discovered in subgroups RL and RF. Detailed univariate analysis is summarized in **Supplemental Table 2**.

**Table 2:** Multivariate analysis of prognostic factors for initial recurrence or progression.

R/R PCNSL			
Factors	HR	95% CI	p
Deep lesions	1.429	0.913-2.237	0.118
Male	0.790	0.519-1.202	0.270
Induction-RT	1.558	0.869-2.793	0.137
Ocular lymphoma	0.708	0.235-2.136	0.540
LDH>250U/L	1.559	0.796-3.053	0.195
RL group			
Factors	HR	95% CI	p
≥60y	0.516	0.195-1.366	0.183
Deep lesions	1.387	0.624-3.081	0.422
CSF protein>0.45g/L	0.438	0.177-1.083	0.074
LDH>250U/L	0.270	0.051-1.425	0.123
RF group			
Factors	HR	95% CI	p
Male	0.654	0.393-1.091	0.104
Induction-RT	3.019	0.929-9.807	0.066

*All factors were measured at diagnosis.

### Response of salvage therapy

The distribution of salvage therapy received was shown in **Supplemental Table 3**. In the RT group, one relapsed patient received stereotactic radiosurgery (SRS) while the other patients received whole brain radiotherapy (WBRT). Among patients received WBRT with detailed record, 12 received a total dose of 20-30 Gy (5 in RL group and 7 in RF group), 4 received a total dose of 36-48 Gy (2 in RL group and 2 in RF group), in fractionation of 1.8 to 2 Gy. In the CT group, treatment programs included HD-MTX reuse, rituximab, idarubicin, cytarabine, Bruton’s tyrosine kinase inhibitors and et al. (**Supplemental Figure 2**). No significant difference was observed in therapy strategy choice (*χ^2^ = *3.184, *p* = 0.203).

**Table 3** shows the clinical efficacy in enrolled subjects with R/R PCNSL. Five losing patients were excluded in this section but were included in the analysis at the last follow-up. The rate of CR was 35% (14 of 40) in the RL group, with 18.3% (11 of 60) in the RF group. The objective response rates (ORR) were 52.5% and 36.7% for RL and RF groups, respectively (*p* = 0.043).

**Table 3:** Response of salvage therapy in R/R PCNSL.

	RL (%)	RF (%)	All (%)	*z*	*p*
CR	35.0	18.3	25.0	-2.021	0.043
PR	17.5	18.3	18.0		
SD	10.0	6.3	8.0		
PD	35.7	56.7	49.0		
ORR	52.5	36.7	43.0		

Furthermore, subgroup analysis of the effect of treatment strategy on the near-term outcomes (**Supplemental Table 4**) showed increased ORR in radiotherapy with or without adjuvant chemotherapy as compared with chemotherapy only, either in RL or RF group (*p* < 0.05).

### Outcome and survival after recurrence and progression

The present analysis was done on data frozen on 31 May 2021. Three patients in the RF group were not followed up to a second progressive or relapsed outcome but only to a survival outcome. Totally 10 patients were lost to follow-up about the end-point, including 3 from the RL group and 7 from the RF group. The median follow-up for 95 patients was 17.5 months (range, 2.5m to 65.7m). **Table 4** shows 15 (14.7%) patients with re-recurrence after obtaining CR with salvage therapy, 69 (67.6%) patients with progression, and 18 (17.6%) patients without events. A total of 41 patients died as a result of advanced tumors or severe complications. The event rate and death rate were similar in RL and RF groups: 81.0% vs. 83.3%, *p*=0.796; 38.1% vs. 41.7%, *p* =0.747, respectively.

**Table 4:** End-point of R/R PCNSL after salvage therapy.

	RL (n)	RF (n)	n/N (%)	χ^2^	p
Primary end-point					
recurrence/progression	9/25	6/44	15/69(82.4)	0.096	0.796
Non-event	8	10	18(17.6)		
Secondary end-point					
Alive	23	31	54(56.8)	0.584	0.747
Death	16	25	41(43.2)		

**Figure 3:**
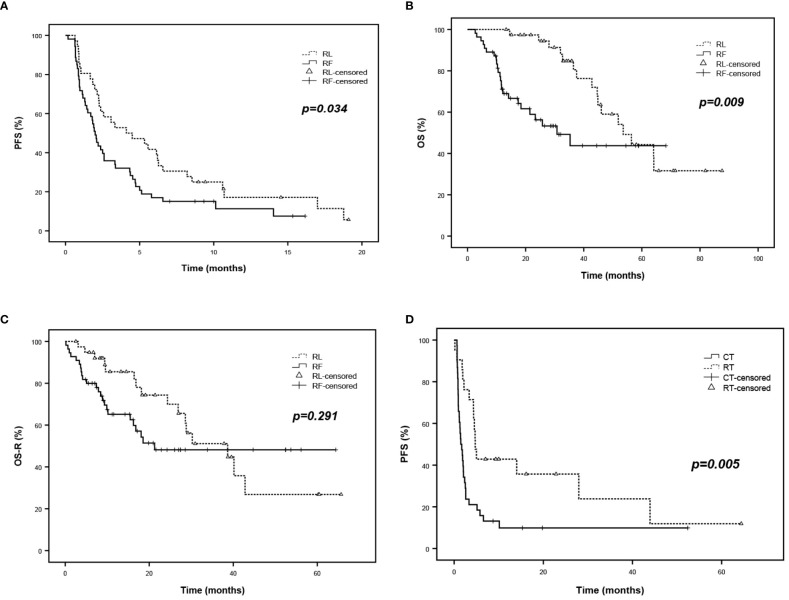
Survival analysis of R/R PCNSL. **(A)** PFS of R/R PCNSL patients after salvage treatment according to RL and RF group. **(B)** OS of R/R PCNSL patients according to RL and RF group. **(C)** OS-R (OS after salvage treatment) of R/R PCNSL patients according to RL and RF group. **(D)** PFS of refractory PCNSL patients after salvage treatment according to RT and CT subgroup.

Among R/R PCNSL patients, median progression-free survival (PFS) was 3.1 months (*95%CI*, 1.3 to 4.8), which was 5.3 months (*95%CI*, 2.2 to 8.5) in the RL group and 2.2 months (*95%CI*, 1.6 to 2.8) in RF group *(p*=0.034) (**Figure 3A**), respectively.

The median overall survival (OS) was 46 months (*95%CI*, 35.1 to 57.0) among all enrolled patients, 53.6 months (*95%CI*, 39.3 to 67.8) in the RL group and 30.8 months (*95%CI*, 15.8 to 45.8) in RF group (*p* = 0.009) (**Figure 3B**), respectively. However, after removing the effect of PFS1 on OS, no significant difference was observed in the median OS after salvage therapy between the RL group and the RF group (38.7 months vs. 21.3 months, *p*=0.291) (**Figure 3C**).

Besides, relapse patients in the RT group showed inferior PFS compared to those in the CT group. The PFS rates were 40.0% vs. 13,5% at 6 months (*p* = 0.005) (**Figure 3D**) (**Supplemental Figure 1**).

### Prognostic factors of R/R PCNSL

**Figure 4:**
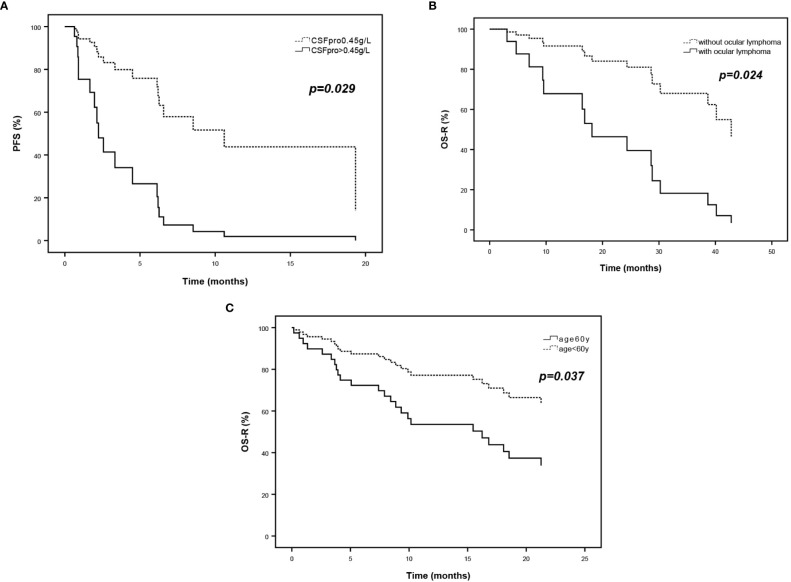
Survival curves of multivariate analysis of prognostic factors for relapsed and refractory PCNSL. **(A)** PFS curves according to CSF protein and **(B)** OS-R curves according to ocular lymphoma of relapsed PCNSL. **(C)** OS-R curves according to age at diagnosis of refractory PCNSL.

**Table 5** shows the multivariate analysis of prognostic factors for relapsed and refractory PCNSL related to the progression of disease and death. The HR for re-progression of relapsed PCNSL, adjusted for major prognostic factors, was 3.531 for CSF protein > 0.45g/L compared to ≤0.45g/L (*95%CI*, 1.141 to 10.922, *p* = 0.029). The HR for death of relapsed PCNSL was 4.415 for ocular involvement at recurrence compared to non-ocular involvement(*95%CI*, 1.221 to 15.957, *p* =0.024), which of refractory PCNSL was 2.535 for age ≥60years compared to <60 years (*95%CI*, 1.060 to 6.066, *p* =0.037) (**Figure 4**).

**Table 5:** Multivariate analysis of prognostic factors for relapsed and refractory PCNSL.

RL group								
	PFS				OS-R			
Factors	HR	95% CI	p	Factors	HR		95% CI	p
≥60y	0.702	0.269-1.834	0.471	Male	4.312		0.876-21.219	0.072
KPS<70	0.742	0.293-1.880	0.529	≥60y	1.076		0.324-3.582	0.904
CSF protein>0.45g/L	3.531	1.141-10.922	0.029	KPS<70	0.629		0.166-2.383	0.495
CSF cells>8×10^6^/L	0.825	0.205-3.316	0.787	Ocular lymphoma	4.415		1.221-15.957	0.024
				PFS1<12m	2.342		0.603-9.089	0.219
RF group								
	PFS					OS-R		
Factors	HR	95% CI	p	Factors		HR	95% CI	p
≥60y	2.302	0.151-1.246	0.121	Male		1.942	0.797-4.732	0.144
KPS<70	1.058	0.342-3.273	0.921	≥60y		2.535	1.060-6.066	0.037
Deep lesions	1.089	0.364-3.255	0.879	KPS<70		1.696	0.713-4.035	0.232
LDH >250U/L	1.117	0.361-3.454	0.848	PFS1<6m		1.743	0.620-4.900	0.292
CSF protein>0.45g/L	1.352	0.392-4.659	0.633	LDH >250U/L		1.178	0.422-3.291	0.755
CSF cells>8×10^6^/L	2.263	0.689-7.438	0.179					

*KPS, CSF protein, CSF cells, and ocular lymphoma were measured at recurrence.

*KPS, LDH, CSF protein, CSF cells, and ocular lymphoma were measured at progression.

## Discussion

PCNSL, as a highly heterogeneous hematologic malignancy with high aggressiveness, easy recurrence, and poor prognosis, has always been a hot spot of concern in the field of hematology. Newly diagnosed PCNSL with solid pathology diagnosis at our institution accepted high-dose MTX with or without rituximab as initial therapy, or whole brain radiotherapy while accompanied with contraindication of encephalic biopsy or chemotherapy. However, somatic disorders caused by the nervous system and ocular involvement greatly affect the patient’s normal social role and reduce the quality of life, especially in the population of R/R PCNSL patients. Therefore, the exploration of effective salvage treatment for R/R PCNSL and the delay of disease progression are major challenges in clinical work.

Most of the previously conducted clinical studies related to R/R PCNSL have studied either recurrent PCNSL or refractory PCNSL (14), or have discussed both patients as a whole (15, 16). However, our results revealed the heterogeneity between the two groups of patients. According to the definition in this paper, recurrent PCNSL could achieve CR after induction therapy, whereas refractory PCNSL fails to achieve PR or even develops PD early. Obviously, the differences in PFS1 indicate that the response to induction therapy differed significantly between relapsed and refractory patients. As the choice of salvage treatment options was not different between RL and RF groups, the difference in response to salvage treatment instead emerged with a significantly higher ORR in the relapsed PCNSL. It can be hypothesized that after the initial relapse/progression, relapsed PCNSL remains higher sensitive to salvage therapy than refractory PCNSL. However, specific mechanisms remain to be explored.

According to the results of previous studies, the median PFS of PCNSL after relapse/progression is only 2-5 months (17–19), which is consistent with our results (3.1 m of all patients, 5.3m of RL, 2.2m of RF). With the limitations of retrospective analysis, the treatment regimen showed high heterogeneity. We briefly divided patients who received salvage chemotherapy into MTX-based group and non-MTX group, no significance was observed. Furthermore, Ferreri and colleagues reported a new chemoimmunotherapy, MATRix, with higher complete remission rate (49%) compared with methotrexate-cytarabine alone (23%) or plus rituximab (30%), which encourage newly combination to apply in newly diagnosed PCNSL and relapsed/refractory PCNSL (20).

Radiotherapy is often considered as consolidation therapy or deferred until relapse. Further analysis of the efficacy of the salvage regimen in R/R PCNSL in this article revealed that the response rate for radiotherapy was significantly higher than that for chemotherapy in both the relapse and refractory groups. In refractory PCNSL, radiotherapy also resulted in a longer duration of disease remission. Another retrospective cohort study showed that salvage WBRT results in longer PFS and higher CR rates compared with high-dose cytarabine, with 10 months of median PFS and 54% for 1-year OS rate (21). The safety and efficacy of salvage WBRT had been evaluated by Hottinger, showing 79% for response rate (22). Furthermore, the occurrence of delayed neurotoxic events may also be greatly reduced with unimpaired disease control (5-year failure-free survival, 51% vs. 50%) when the total WBRT dose is controlled to less than 36Gy according to Ferreri and the International Extranodal Lymphoma Study Group (23, 24). A case series from National Cancer Institute of Colombia also supports the benefit of radiotherapy with effective local control and long term survival up to 10 years (25). However, radiotherapy did not show a significant advantage in OS in this paper, although numerically the median post-relapse/progression OS was longer in the CT group than in the RT group. These results suggest that we can use radiotherapy in the early stages of relapse/progression as an access to delay progression in the short term, improve patients’ quality of life to some extent, and gain the opportunity for patients to try more treatments. However, further studies are needed to design better treatment strategies to give patients the benefit of long-term survival.

To predict prognosis as accurately as possible and select more appropriate treatment options, new prognostic factors need to be explored. In this study, we verified that age ≥60 years at diagnosis was an independent adverse prognostic factor for OS after recurrence/progression, which is consistent with other studies and grading criteria (26–28). Besides, cox regression analysis shows patients with abnormally elevated CSF protein are more likely to undergo progression. It has been suggested that cerebrospinal fluid cells and protein levels are important prognostic assessment factors (29, 30) because they both reflect the extent of meningeal involvement and intracranial tumor load to some extent. But in this paper results were not matched in univariate and multivariate analyses, caused by patients admitted to other hospitals for treatment without administering CSF examination in our institution.

The results of our previous study (31) showed that patients with concomitant intraocular lymphoma were more likely to relapse compared to patients without intraocular lymphoma (relapse rates, 71.4% vs. 46.3%), whereas in this study we found that concomitant intraocular involvement at the time of relapse was associated with shorter post-recurrence OS. Survival could be affected when patients accepted intraocular MTX injection, which is also a treatment regimen adjustment. Intraocular lymphoma is an important branch of PCNSL, and clinicians can continue to explore the relationship between intraocular involvement and survival in multidisciplinary collaboration with the ophthalmology department.

In conclusion, radiotherapy could be a viable salvage treatment option for R/R PCNSL patients with initial recurrence or progression, demonstrating better antitumor effects and allowing for longer disease remission, at least in the early stages. Age, ocular involvement, and level of CSF protein may serve as potential prognostic predictors. However, multicenter, large-sample, and prospective studies are still needed to explore who benefits more in overall survival with radiotherapy versus chemotherapy after relapse/progression.

## Data availability statement

The original contributions presented in the study are included in the article/**Supplementary Material**. Further inquiries can be directed to the corresponding authors.

## Ethics statement

The studies involving human participants were reviewed and approved by the ethical review boards of Huashan Hospital, Fudan University. The patients/participants provided their written informed consent to participate in this study.

## Author contributions

YY, YM, and BC designed the research; YY acquired patients, analyzed data, and wrote the manuscript; YM and BC reviewed and revised the manuscript. QL, JM, HK, and ZL participated in the implementation of chemotherapy treatment. YW participated in the implementation of radiotherapy. All authors contributed to the article and approved the submitted version.
