# Supplementary material for: Radiotherapy or chemotherapy: a real-world study of the first-time relapsed and refractory primary central nervous system lymphoma
Source: Front Oncol. 2023 Apr 27;13:1098785. doi: 10.3389/fonc.2023.1098785 (PMC10174451; doi:10.3389/fonc.2023.1098785)
Supplement: Supplementary file 1 [file DataSheet_1.docx]

Supplemental Table 1 Distribution of induction treatment received.

| treatment | All (*n*) | RL (*n*) | RF (*n*) | *χ^2^* | *p* |
| --- | --- | --- | --- | --- | --- |
| CT | 91 | 32 | 59 | 6.678 | 0.035 |
| MTX-based | 90 | 31 | 59 |  |  |
| Non-MTX | 1 | 1 | 0 |  |  |
| RT | 4 | 3 | 1 |  |  |
| RT+CT | 10 | 7 | 3 |  |  |

Supplemental Table 2 Univariate analysis of prognostic factors for initial recurrence or progression at diagnosis

| Factors | RL | | RF | | All | |
| --- | --- | --- | --- | --- | --- | --- |
|  | *χ^2^* | *p* | *χ^2^* | *p* | *χ^2^* | *p* |
| Gender | 0.118 | 0.731 | 1.937 | 0.164 | 1.711 | 0.191 |
| Age | 3.525 | 0.060 | 0.014 | 0.905 | 0.017 | 0.895 |
| Induction therapy | 0.149 | 0.699 | 3.175 | 0.075 | 2.656 | 0.103 |
| Biopsy | 0.492 | 0.483 | 1.149 | 0.234 | 1.544 | 0.214 |
| Deep lesions | 1.926 | 0.165 | 0.005 | 0.943 | 4.616 | 0.032 |
| Lesion numbers | 0.084 | 0.772 | 0.958 | 0.620 | 1.384 | 0.501 |
| Ocular lymphoma | - | -^**^ | 0.006 | 0.941 | 2.840 | 0.092 |
| KPS | 0.574 | 0.449 | 1.371 | 0.242 | 1.447 | 0.229 |
| LDH | 2.006 | 0.157 | 0.000 | 0.999 | 4.271 | 0.039 |
| CSF cells | 0.006 | 0.938 | 0.020 | 0.887 | 0.321 | 0.571 |
| CSF protein | 10.637 | 0.001 | 0.901 | 0.343 | 0.560 | 0.454 |

*Factors with p < 0.2 were enrolled in multivariate analysis.

**None of the RL group had ocular lymphoma at diagnosis.

Supplemental Table 3 Distribution of salvage treatment received

| treatment | All (*n*) | RL (*n*) | RF (*n*) | *χ^2^* | *p* |
| --- | --- | --- | --- | --- | --- |
| CT | 67 | 26 | 41 | 3.184 | 0.203 |
| MTX-based | 33 | 13 | 20 |  |  |
| Non-MTX | 34 | 13 | 21 |  |  |
| RT | 31 | 11 | 20 |  |  |
| RT+CT | 7 | 5 | 2 |  |  |

Supplemental Table 4. ORR in R/R PCNSL referred to different salvage therapy

|  | RL | | | RF | | |
| --- | --- | --- | --- | --- | --- | --- |
|  | ORR(%) | *z* | *p* | ORR(%) | *z* | *p* |
| ① |  |  |  |  |  |  |
| RT, RT+CT | 78.6 | -2.723 | 0.014 | 61.9 | -3.535 | <0.01 |
| CT | 38.5 |  |  | 23.1 |  |  |
| ② |  |  |  |  |  |  |
| RT | 88.9 | -2.296 | 0.04 | 57.9 | -2.936 | <0.01 |
| CT, CT+RT | 41.9 |  |  | 26.8 |  |  |
| ③ |  |  |  |  |  |  |
| RT | 88.9 | -2.470 | 0.028 | 57.9 | -3.236 | <0.01 |
| CT | 38.5 |  |  | 23.1 |  |  |

Supplementary Figure 1

Survival analysis of relapsed or refractory PCNSL according to RT and CT subgroup.

(a) PFS, (b) OS, and (c) OS-R of relapsed PCNSL patients according to RT and CT subgroup. (d) OS and (e) OS-R of refractory PCNSL patients according to RT and CT subgroup.

Supplementary Figure 2

Detailed chemotherapy regiment (A) and radiotherapy regiment (B) of relapsed and refractory PCNSL.
