# Supplementary figures and images for: Radiotherapy or chemotherapy: a real-world study of the first-time relapsed and refractory primary central nervous system lymphoma
Source: Front Oncol. 2023 Apr 27;13:1098785. doi: 10.3389/fonc.2023.1098785 (PMC10174451; doi:10.3389/fonc.2023.1098785)

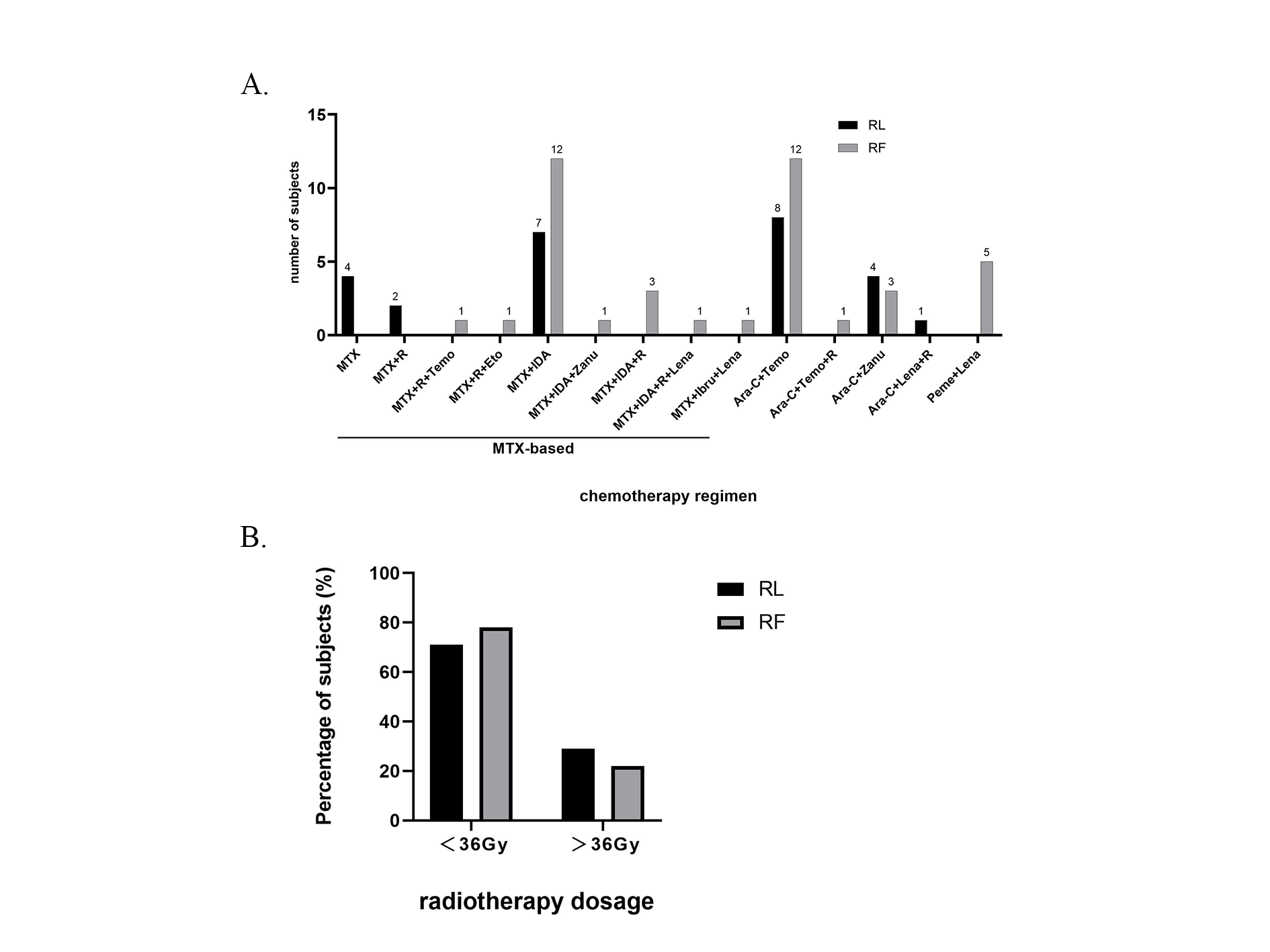

Supplement: Supplementary file 3 [file Image_2.tif]
